# Visual Complexity and Affect: Ratings Reflect More Than Meets the Eye

**DOI:** 10.3389/fpsyg.2017.02368

**Published:** 2018-01-18

**Authors:** Christopher R. Madan, Janine Bayer, Matthias Gamer, Tina B. Lonsdorf, Tobias Sommer

**Affiliations:** ^1^Department of Systems Neuroscience, University Medical Center Hamburg-Eppendorf, Hamburg, Germany; ^2^School of Psychology, University of Nottingham, Nottingham, United Kingdom; ^3^Department of Psychology, University of Würzburg, Würzburg, Germany

**Keywords:** visual complexity, affect, arousal, valence, eyetracking, emotion

## Abstract

Pictorial stimuli can vary on many dimensions, several aspects of which are captured by the term ‘visual complexity.’ Visual complexity can be described as, “a picture of a few objects, colors, or structures would be less complex than a very colorful picture of many objects that is composed of several components.” Prior studies have reported a relationship between affect and visual complexity, where complex pictures are rated as more pleasant and arousing. However, a relationship in the opposite direction, an effect of affect on visual complexity, is also possible; emotional arousal and valence are known to influence selective attention and visual processing. In a series of experiments, we found that ratings of visual complexity correlated with affective ratings, and independently also with computational measures of visual complexity. These computational measures did not correlate with affect, suggesting that complexity ratings are separately related to distinct factors. We investigated the relationship between affect and ratings of visual complexity, finding an ‘arousal-complexity bias’ to be a robust phenomenon. Moreover, we found this bias could be attenuated when explicitly indicated but did not correlate with inter-individual difference measures of affective processing, and was largely unrelated to cognitive and eyetracking measures. Taken together, the arousal-complexity bias seems to be caused by a relationship between arousal and visual processing as it has been described for the greater vividness of arousing pictures. The described arousal-complexity bias is also of relevance from an experimental perspective because visual complexity is often considered a variable to control for when using pictorial stimuli.

## Introduction

Berlyne (1958) described visual complexity as being influenced by a variety of factors, including number of the comprising elements, as well as their heterogeneity (e.g., a single shape repeated vs. multiple distinct shapes), their regularity (e.g., simple polygons vs. more abstract shapes) and the regularity of the arrangement of elements (e.g., symmetry, distribution characteristics) (see Figure 1 of Berlyne, 1958). More recently, Pieters et al. (2010) provided a similar list of complexity factors, along with example advertisements that highlight these differences (Oliva et al., 2004; see Figure 2 of Pieters et al., 2010). Research into visual complexity has recently become a multidisciplinary topic, involving researchers in fields ranging from marketing (Pieters et al., 2010; Braun et al., 2013) to computer science (Itti et al., 1998), and from esthetics (Nadal et al., 2010) to human–computer interaction (Tuch et al., 2009) as well as psychology (Donderi, 2006; Cassarino and Setti, 2015, 2016). This approach to examining visual complexity in pictures that are clearly and consciously viewed, and judged based on the number of objects and their aggregate structure, is the focus of the current work.

Many studies have observed a relationship between visual complexity and affect (e.g., pleasantness). This relationship has been observed dating back to the early 1970s (e.g., Kaplan et al., 1972; Aitken, 1974; Aitken and Hutt, 1974) and this idea has re-emerged more recently (e.g., Stamps, 2002; Marin and Leder, 2013, 2016; Schlochtermeier et al., 2013; Machado et al., 2015; Marin et al., 2016). Importantly, these studies suggest that more complex pictures are perceived as more pleasant than less complex pictures, a hypothesis supported by earlier work where pleasantness and physiological arousal have been found to be higher for more complex abstract shapes (e.g., Berlyne et al., 1963, 1968; Vitz, 1964; Day, 1967). At particularly high levels of complexity, pleasantness decreases, however, following an inverted-U shaped function (“Wundt curve,” Berlyne, 1970). Importantly, in these studies, the directionality of this relationship between complexity and affect is always discussed as complexity influencing affect. However, there might be also an effect in the opposite direction, i.e., affect influencing perceived visual complexity.

This hypothesis seems plausible because emotionally arousing stimuli attract bottom-up attention, are processed with priority as well as a higher signal-to-noise ratio and are perceived more vividly (Pourtois et al., 2013; Markovic et al., 2014; Mather et al., 2016). The relationship between arousal and perception is reflected in greater activity in visual regions as well as in different eye movement patterns (Pessoa and Adolphs, 2010; Bradley et al., 2011; Ni et al., 2011). This difference in visual processing activity has primarily been attributed to autonomic activity and motivational salience. In addition, emotionally arousing stimuli possess certain cognitive characteristics that might influence experienced complexity. First, emotionally arousing stimuli are more distinctive relative to prior experiences (Schmidt, 1991). Second, they are semantically related, i.e., often belong to the same scripts such as disease, poverty, or crime (Talmi and McGarry, 2012). During processing of such a stimuli the associated script might more easily get activated and influence experienced complexity. Taken together, from a basic science perspective there is good evidence to hypothesize that emotionally arousing stimuli are perceived as more complex (Marin and Leder, 2013).

From an experimental approach, visual complexity is often considered a variable to control for when using pictorial stimuli to investigate affective (e.g., Ochsner, 2000; Talmi et al., 2007; Sakaki et al., 2012) or memory processes (e.g., Snodgrass and Vanderwart, 1980; Berman et al., 1989; Isola et al., 2014; Nguyen and McDaniel, 2015). As we were particularly interested in potential top–down effects of affect within studies of affect or memory, we presented pictures for several seconds, as opposed to other studies where pictures may only be presented briefly (e.g., 50–200 ms) along with visual masks. Importantly, visual complexity is often measured through ratings provided by participants in initial norming studies. However, while many studies have matched stimuli using visual complexity ratings, these studies did not consider that ratings of visual complexity may themselves be correlated with affective ratings (i.e., arousal and valence), and thus controlling for ratings of visual complexity might bias the affective quality of the stimulus material.

### Computational Measures of Visual Complexity

To measure visual complexity, computational approaches can also be used. One of the simplest and most prevalent methods used to computationally measure visual complexity is to simply use the picture’s file size after using JPEG compression. The general idea behind this approach is that more complex pictures, given the same picture dimensions, can be compressed to a lesser degree than less complex pictures and thus more complex pictures have larger file sizes. Using this rationale, a number of studies and review articles have suggested the use of JPEG file size as a computational measure of visual complexity (e.g., Machado and Cardoso, 1998; Székely and Bates, 2000; Donderi, 2006; Forsythe et al., 2008, 2011; Martinovic et al., 2008; Forsythe, 2009; Tuch et al., 2009; Pieters et al., 2010; Stickel et al., 2010; Purchase et al., 2012; Marin and Leder, 2013; Schlochtermeier et al., 2013; Simola et al., 2013; Machado et al., 2015). While the JPEG file size does correlate with visual complexity, for scientific research it seems more appropriate to use a method designed to be similar to the computational processes that occur in early visual cortices. Nonetheless, here we will also evaluate the efficacy of JPEG file size as a computational measure of visual complexity.

**FIGURE 1:**
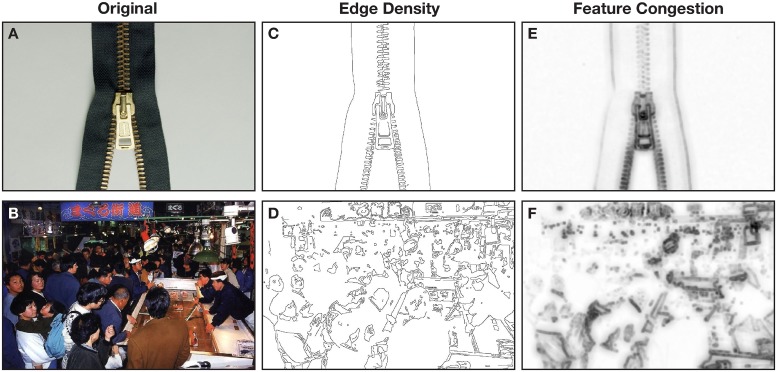
Example pictures shown to participants as exemplars of **(A)** low visual complexity and **(B)** high visual complexity. **(C,D)** Illustrate the edge density of the two pictures. **(E,F)** Illustrate the feature congestion of the two pictures.

One such computational process is edge detection, which is the identification of boundaries within a picture. Though several edge detection algorithms have been developed (for reviews, see Nadernejad et al., 2008; Juneja and Sandhu, 2009; Maini and Aggarwal, 2009), Canny’s (1986) algorithm has been found to generally be better able to detect edges (Nadernejad et al., 2008; Juneja and Sandhu, 2009; Maini and Aggarwal, 2009; Machado et al., 2015) and has been used in behavioral research (e.g., Rosenholtz et al., 2007; Forsythe et al., 2008; Ptak et al., 2009; Sakaki et al., 2012; Machado et al., 2015). See **Figures 1C,D** for examples of edge detection applied to naturalistic pictures^1^. Edge detection can be summarized as ‘edge density,’ where an edge detection map is averaged to calculate a single value corresponding to the proportion of the map was identified as an edge.

Based on prior work developing computational approaches to measure visual complexity (Rosenholtz et al., 2007), we additionally used two other computational measures: feature congestion and subband entropy. Feature congestion quantifies how ‘cluttered’ a picture is and incorporates color, luminance contrast, and orientation. See **Figures 1E,F** for examples of Rosenholtz et al.’s (2007) feature congestion algorithm applied to naturalistic pictures. Subband entropy quantifies the ‘organization’ within the picture, through Shannon’s entropy in spatial repetitions of hue, luminance, and size (i.e., spatial frequency). Rosenholtz et al. (2007) found that all three measures (edge density, feature congestion, and subband entropy) correlated with response time in a visual search task, demonstrating that these three computational measures relate to behavior.

It is worth considering, however, that the computational measures of visual complexity described here are limited to low-level visual features. As such, they would provide similar complexity values for a picture of dozens of leaves as for a picture of dozens of unique toys—whereas a viewer may know that the toys represent characters from different cartoon shows and are associated with more varied semantic information. Ratings of visual complexity are based on both low-level features in addition to high-level features such as object information. Nonetheless, higher-level visual features are difficult to systematically characterize (i.e., using computational algorithms and without subjective biases) and the current work focused on the relationships between computational measures of visual complexity and affective measures with ratings of complexity.

In sum, visual complex stimuli are perceived as more (positive) emotionally arousing. Further, it is well established that emotionally arousing stimuli attract selective attention, alter sensory processing and are reported as having higher vividness which might translate into higher experienced visual complexity (Marin and Leder, 2013). Such a greater visual complexity of emotionally arousing stimuli might also be supported by differences in cognitive processing, i.e., their higher distinctiveness and semantic relatedness. Based on these considerations we aimed to characterize the relationship between emotional arousal and perceived visual complexity in the current study.

The hypothesized relationship between emotional arousal and experienced visual complexity is also relevant from an experimental perspective because ratings are widely used to generate equally complex picture sets that differ only with respect to arousal and valence. Therefore, we investigated the relationship between affective properties of scenic stimuli, arousal and valence, and computational measures of visual complexity on experienced visual complexity (i.e., participant ratings). In doing so, the four most often proposed computational measures of visual complexity were compared as a secondary outcome. Admittedly, here we did not manipulate the pictures themselves and investigated the correlational relationship between affect and perceived visual complexity, rather than attempting to causally influence this relationship.

In a series of experiments, we examined the contributions of affective processes and computational measures of visual complexity to visual complexity ratings in naturalistic pictures. After establishing this effect across different subsets of stimuli, rating procedures, and presentation times (Experiments 1–2) we further explored how ratings of visual complexity related to measures of cognition, eye-tracking, emotion-related traits and deliberate control (Experiments 3–5).

## Experiment 1

In Experiment 1, we first tested for relationships between affective and visual complexity ratings, as well as for relationships with the computational measures of visual complexity, across a large set of 720 pictures. In addition, the four computational measure of visual complexity—edge density, feature congestion, sub band entropy, and JPEG file size—were formally compared with respect to the shared variance with the ratings of visual complexity.

### Methods

#### Participants

As prior studies have indicated that there are likely sex differences in affective processing (Cahill et al., 2001; Canli et al., 2002; Sergerie et al., 2008; Schneider et al., 2011; Brown and Macefield, 2014), we only recruited female participants in all experiments. In addition, we restricted the sample to female volunteers to improve inter-rate consistency, particularly since some positively valenced, arousing stimuli were erotic in nature. A total of 35 female volunteers (ages 18–40) with normal or corrected-to-normal vision participated. In all experiments, volunteers were recruited through an advertisement on the homepage of the University of Hamburg, gave informed written consent, and received monetary reimbursement (10€ per hour) for their participation. No volunteer participated in more than one experiment. The research was approved by the local ethics board (Board of Physicians, Hamburg, Germany).

#### Materials

A total of 720 pictures were used in the experiment: 239 pictures were selected from the International Affective Picture System (IAPS; Lang et al., 2008) database, and were supplemented by an additional 481 pictures found on the Internet that were thematically similar to pictures in the IAPS (and were adjusted to have the same picture dimensions as the IAPS pictures).

Pictures were chosen such that the picture set was approximately one-third each of positive, negative, and neutral pictures. Importantly, pictures were chosen such that pictures were distributed across six categories with different numbers of primary objects in the foreground (objects, animals, faces, one-person scenes, two-person scenes, multi-person scenes). Pictures in each topic category were evenly distributed across the three valences.

#### Procedure

**FIGURE 2:**
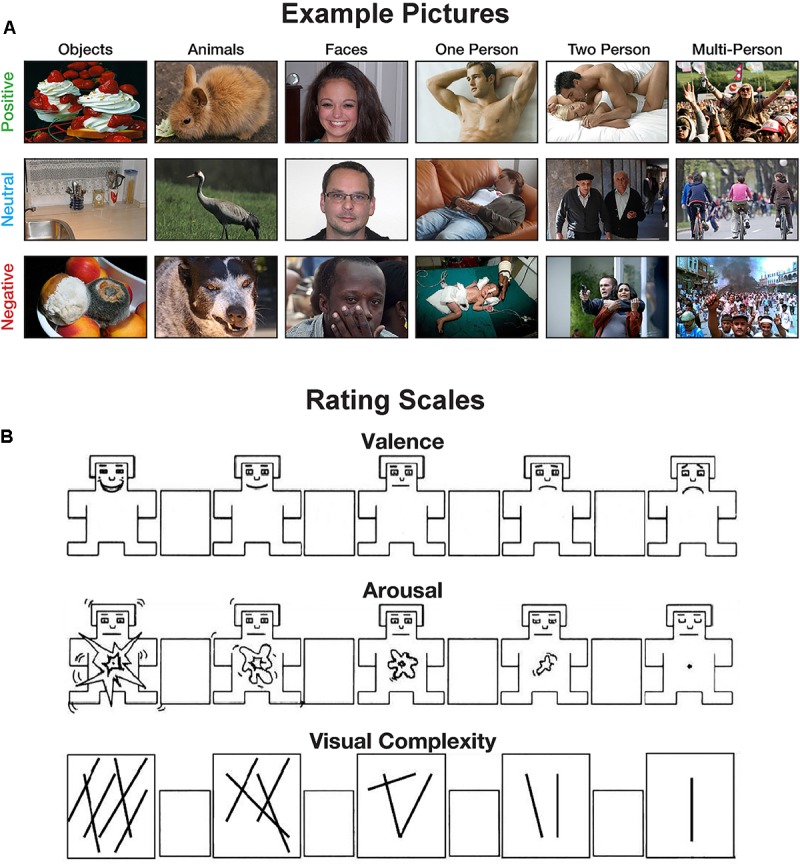
Examples of the experimental methods. **(A)** Example pictures used in the experiments. **(B)** Scale used for the valence, arousal, and visual complexity ratings. The valence and arousal scales are adapted from the self-assessment manikin (SAM) developed by Bradley and Lang (1994).

Participants were told that they will be shown emotional pictures and be asked to rate these pictures on three scales: valence, arousal, and visual complexity. Participants were provided with instructions describing each measure. For the valence and arousal ratings, instructions were identical to those used by Lang et al. (2008) and participants rated the pictures using the 9-point Self-Assessment Manikin (SAM; Bradley and Lang, 1994). For the visual complexity rating, participants were instructed that: “A picture of a few objects, colors, or structures would be less complex than a very colorful picture of many objects that is composed of several components.” To further orient participants to this type of rating, participants were provided two example pictures: one low-complexity picture (**Figure 1A**) and one high-complexity picture (**Figure 1B**). A 9-point Likert scale, shown in **Figure 2**, was used for complexity ratings. For ratings of complexity and arousal, the left-most options corresponded to higher ratings of complexity and arousal, respectively. For valence, left-most options corresponded with higher ratings of pleasantness, lower ratings corresponded with unpleasantness.

On each trial, participants were first shown a picture for 2000 ms, followed by the rating screen, which persisted until all three ratings were given using the computer mouse. The order of ratings was constant across all trials and participants: valence, arousal, visual complexity.

Over two consecutive days, participants rated all 720 pictures for valence, arousal, and visual complexity (360 pictures per day; 1 h per day). An additional 5 ‘buffer’ pictures were presented as the first trials on the first day, to allow participants to become accustomed to the task.

#### Data Analysis

Effects were considered significant based on an alpha level of 0.05. Ratings of visual complexity, valence and arousal were computed as averages across participants to obtain normative ratings for each picture, as some of the experiments only involved a subset of the rating scales.

To examine the relative relationships of the examined measures with ratings of visual complexity, we conducted a hierarchical regression. In this regression, we first evaluated regression models that included only individual measures. Next, we evaluated models that had either affective ratings or computational measures. Finally, we evaluated a ‘full’ model that contained both sets of measures. The list of models considered and their respective model fitness measures are reported in **Table 1**. In subsequent experiments, we used subsets of the 720 pictures, with either 360 or 144 pictures; to demonstrate the robustness of the observed findings, model fitness indices are reported for these subsets as well. All subsets had an equal number of images from each category. Mean ratings/scores for each measure, for each category, are reported in **Table 2**.

**Table 1:** Hierarchical regression analysis of rated visual complexity with affective ratings and computational measures of visual complexity, across all 720 images used in Experiment 1 and for only the subsets used in Experiments 2–4 (360 and 144 image subsets).

	*Measures*						*Picture Set*					
	Affective ratings		Computational visual complexity				All 720		360 subset		144 subset	
Model	Arousal	Valence	Edge density	Feature congestion	Subband entropy	JPEG file size	*R*^2^	*ΔBIC*	*R*^2^	*ΔBIC*	*R*^2^	*ΔBIC*
**Individual Measures**												
Arousal	X						0.294	250.18	0.328	130.26	0.321	44.68
Valence		X					0.064	452.28	0.075	245.35	0.089	87.02
Edge density			X				0.147	385.82	0.158	211.54	0.181	71.76
Feature congestion				X			0.199	340.84	0.215	186.16	0.211	66.35
Subband entropy					X		0.028	479.80	0.037	259.53	0.024	96.89
JPEG file size						X	0.114	413.53	0.089	239.73	0.054	92.43
							


Affective ratings	X	X					0.298	252.80	0.330	135.00	0.321	49.62
							


Computational visual complexity												
w/ JPEG file size			X	X	X	X	0.235	327.70	0.266	179.81	0.289	66.16
w/o JPEG file size			X	X	X		0.233	322.96	0.249	181.86	0.272	64.78
							


Full Model (w/o JPEG File Size)	X	X	X	X	X		0.524	0.00	0.569	0.00	0.581	0.00

**Table 2:** Mean (*SD*) values for each of the rating and computational measures, for each picture category, from the full set of 720 pictures.

	Picture category					
Measure	Objects	Animals	Faces	One person	Two person	Multi-person
Number of Pictures (out of 720)	120	120	120	120	120	120
**Ratings**						
Visual complexity	3.74 (1.11)	3.92 (0.76)	3.52 (0.43)	4.18 (0.71)	4.63 (0.7)	5.95 (0.72)
Arousal	3.96 (1.11)	4.48 (1.08)	3.8 (0.73)	4.47 (1.31)	5.07 (1.41)	4.77 (1.04)
Valence	4.79 (1.32)	5.24 (1.5)	4.96 (0.96)	4.87 (1.54)	4.66 (1.61)	4.84 (1.45)
**Computational visual complexity**						
Edge density	0.0405 (0.0253)	0.0532 (0.0340)	0.0208 (0.0119)	0.0268 (0.0211)	0.0329 (0.0242)	0.0581 (0.0241)
Feature congestion	3.31 (0.90)	3.52 (1.18)	2.35 (0.52)	2.75 (0.64)	3.07 (1.00)	4.24 (1.32)
Subband entropy	3.63 (0.37)	3.68 (0.45)	3.18 (0.41)	3.43 (0.3)	3.48 (0.39)	3.62 (0.34)

For each regression model, we report both *R*^2^, with ratings of visual complexity as the dependent measure, and *ΔBIC*. This second fitness index is the Bayesian Information Criterion (*BIC*), which includes a penalty based on the number of free parameters. Smaller *BIC* values correspond to better model fits. By convention, two models are considered equivalent if *ΔBIC* < 2 (Burnham and Anderson, 2004). As *BIC* values are based on the relevant dependent variable, *ΔBIC* values are reported relative to the best-performing model (i.e., *ΔBIC* = 0 for the best model).

### Results and Discussion

#### Individual Regression Models

##### Arousal and valence

**FIGURE 3:**
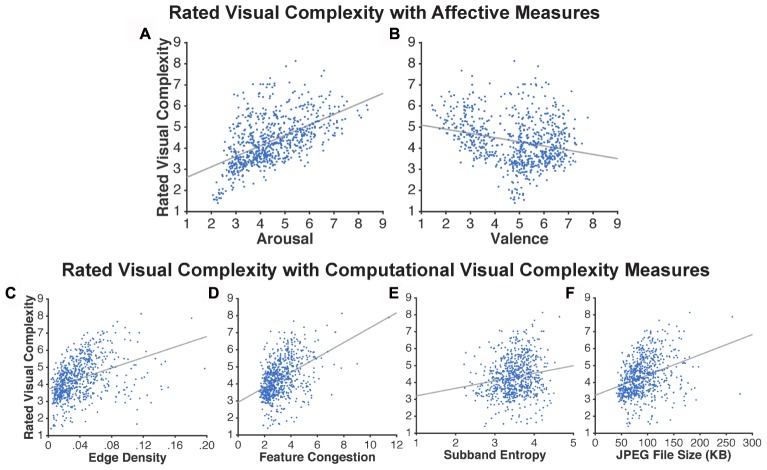
Scatter plots based on the ratings obtained in Experiment 1. Relationship between visual complexity and affect ratings: **(A)** arousal and **(B)** valence. Relationship between visual complexity ratings and computational measures: **(C)** edge density, **(D)** feature congestion, **(E)** subband entropy, and **(F)** JPEG file size. Each dot represents an individual picture (720 in total); lines represent linear regressions.

As shown in **Figure 3** and **Table 1**, arousal was more related to ratings of visual complexity than valence [arousal: *R*^2^ = 0.294; valence: *R*^2^ = 0.06].

##### Computational measures of visual complexity

Visual complexity was computationally measured using edge density, feature congestion, and subband entropy. The three measures of visual complexity were significantly correlated with each other [edge density ↔ feature congestion: *r*(718) = 0.78, *p* < 0.001; edge density ↔ subband entropy: *r*(718) = 0.60, *p* < 0.001; feature congestion ↔ subband entropy: *r*(718) = 0.65, *p* < 0.001]. Here we additionally included JPEG file size to test if these three more formal measures of visual complexity are able to account for the variance explained by the JPEG file size (**Figure 3F**).

As shown in **Table 1**, these computational measures were able to explain significant portions of variability in visual complexity ratings, particularly feature congestion [*R*^2^ = 0.199]. However, all of the *R*^2^ values were still lower than ratings of arousal.

As an additional test for the utility in operationalizing visual complexity as JPEG file size, we conducted correlations between it and the other three computational measures of visual complexity. JPEG file size correlated highly with all three measures [edge density: *r*(718) = 0.78, *p* < 0.001; feature congestion: *r*(718) = 0.82, *p* < 0.001; subband entropy: *r*(718) = 0.64, *p* < 0.001].

#### Multiple Regression Models

To better characterize the relationships between these seven measures (arousal, valence, edge density, feature congestion, subband entropy, JPEG file size) on ratings of visual complexity, we conducted a series of multiple regression models within the hierarchical regression framework.

In the first model, we included only affective ratings (arousal, valence) and found that together they accounted for a sizeable portion of the variance in rated visual complexity [*R*^2^ = 0.298]. In the second model, we included only the computational measures of visual complexity (edge density, feature congestion, subband entropy, JPEG file size) and found that together they yielded adjusted *R*^2^ = 0.235. Excluding JPEG file size had a minimal effect on the amount of variance explained (decrease in adjusted *R*^2^ from 0.235 to 0.233). Given this lack of additional variance explained, and the correlations reported above, JPEG file size was excluded from further analyses.

In the last model we included six measures, two of affect ratings (arousal, valence) and three of computational measures of visual complexity (edge density, feature congestion, subband entropy). Here we found that the combined model explained half of the variance in visual complexity ratings [*R*^2^ = 0.524]. Given this incremental approach, it is clear that the affective ratings and computational visual complexity measures each explain unique portions of variance in visual complexity ratings. Nonetheless, we further tested for associations between affective ratings and computational visual complexity measures, and all were found to be non-significant [all *r*’s < 0.1; *p*’s > 0.05].

Taken together, the results of Experiment 1 demonstrated that despite the ratings indicating the contrary, emotional pictures were not more complex when evaluated using computational measures. This implies that complex pictures may not be higher in positive-valenced arousal as suggested in earlier studies (e.g., Berlyne et al., 1963, 1968; Vitz, 1964; Day, 1967; Kaplan et al., 1972; Aitken, 1974; Aitken and Hutt, 1974; Nadal et al., 2010; Forsythe et al., 2011). One potential explanation could be the nature of the employed visual stimuli, e.g., paintings or abstract pictures vs. natural scenes.

Importantly, Experiment 1 shows clearly that the affective factors, arousal and valence, relate to visual complexity ratings independent of the computational measures, where the effect of arousal is much more pronounced, i.e., explains substantially more unique variance. Mechanisms regarding how emotional arousal might enhance not only perception and related experienced vividness (Todd et al., 2012), but also perceived complexity, will be discussed in the general discussion. Nonetheless, as arousal was more strongly related to this bias in ratings of visual complexity than valence; thus, hereafter we will refer to this effect as the ‘arousal-complexity bias.’

## Experiment 2

In Experiment 1, participants were presented with the pictures for a short duration (2000 ms). During this relatively brief period, participants needed to sample the information necessary to evaluate the pictures for arousal, valence, and visual complexity. Under time pressure, searching for potentially relevant picture characteristics is a demanding, goal-directed task under top-down control. It is known that emotionally arousing stimuli preferentially recruit attentional resources, such as in dual-task conditions, resulting in even greater memory advantages relative to neutral stimuli (Kensinger and Corkin, 2004; MacKay et al., 2004; Mather and Sutherland, 2011; Kang et al., 2014; Madan et al., 2017). Therefore, it may be possible that the greater experienced visual complexity for arousing stimuli in Experiment 1 was partly driven by the preferential recruitment of attentional resources. To test this hypothesis, we presented the pictures for 5 s in Experiment 2 to potentially attenuate any such effect.

Additionally, given that participants in Experiment 1 made their ratings in a fixed order, with the affective ratings always preceding the visual complexity rating, it is possible that we unintentionally induced an effect of affect on ratings of visual complexity. In Experiment 2A, we changed the rating procedure such that the ratings were made sequentially, rather than presenting all three rating scales simultaneously (as in Experiment 1). In Experiment 2B, participants were *only* asked to make visual complexity ratings, removing potential confounding effects of being asked to attend to the emotional features of the picture before the complexity rating as well as lowering the demands on information sampling during processing of the pictures.

To evaluate the influence of these potentially confounding factors, we correlated the ratings obtained in each of these experiment, for each picture, with those obtained in Experiment 1. We also report the mean absolute difference between ratings to evaluate the absolute agreement between the experimental procedures.

Since presentation time was increased, we decreased the picture set to prevent the experiment from becoming too long. This was done by randomly selecting 360 pictures from the full set of 720 pictures used in Experiment 1. To ensure that this picture set was representative, we re-calculated the correlations from Experiment 1 using only this picture subset. As shown in **Table 1**, correlations for this subset were comparable to the full stimulus set.

### Methods

#### Participants

A total of 38 female volunteers with normal or corrected-to-normal vision participated (Experiment 2A: *N* = 20; Experiment 2B: *N* = 18). Consent and reimbursement procedures were identical to Experiment 1.

#### Materials

A subset of 360 pictures was chosen from Experiment 1, such that the pictures were equally distributed across the valence and topic categories.

#### Procedure

Participants were given the same instructions as in Experiment 1. On each trial, participants were presented with a fixation-cross overlaid on a mean luminance picture (i.e., the average luminance of the 360 pictures) for 1000 ms. This was done to keep the stimulus presentation identical for the remaining experiments, and in particular for eye-tracking (Experiment 3) where the fixation-cross was necessary. The fixation-cross was followed by the presentation of a picture for 5000 ms. Next, participants were sequentially shown the rating scales for valence, arousal, and visual complexity (in a fixed order), with a 500 ms delay between the presentation between each ratings screen. A 1000 ms inter-trial interval separated the trials. A total of 9 buffer pictures were presented at the beginning of the task. The entire experimental session took approximately 2 h to complete.

The procedure used in Experiment 2B was identical to that of Experiment 2A, except that participants *only* made visual complexity ratings.

### Results and Discussion

In Experiment 2A, all three measures (ratings of visual complexity, arousal, and valence) were highly correlated with the ratings obtained in Experiment 1 [ratings of visual complexity: *r* = 0.96, *M_diff_* = 0.33; arousal: *r* = 0.94, *M_diff_* = 0.35; valence: *r* = 0.95, *M_diff_* = 0.85]. In Experiment 2B, visual complexity was also highly correlated with the ratings from Experiment 1 [*r* = 0.95, *M_diff_* = 0.49]. Furthermore, correlations between visual complexity and affective ratings and computational measures of visual complexity were markedly similar (**Table 3**).

**Table 3:** Correlations of rated visual complexity with affective ratings and computational measures of visual complexity.

Measure	Experiment 2a	Experiments 2b	Experiment 4			Experiment 5a		Experiment 5b	
			Naïve	bias-aware	difference (*Z*)	Phase 2	Phase 3	naïve	bias-aware
**Ratings**									
Visual complexity (Experiment 1)	0.96^∗∗∗^	0.95^∗∗∗^	0.91^∗∗∗^	0.86^∗∗∗^	6.73^∗∗∗^ (↓)	84^∗∗∗^	0.87^∗∗∗^	84^∗∗∗^	0.81^∗∗∗^
Arousal	0.55^∗∗∗^	-	0.27^∗∗∗^	0.16^∗∗^	6.26^∗∗∗^ (↓)	0.45^∗∗∗^	46^∗∗∗^	0.50^∗∗∗^	40^∗∗∗^
Valence	0.29^∗∗∗^	-	-0.24^∗∗∗^	-20^∗∗∗^	2.34^∗^ (↓)	-0.22^∗∗∗^	0.29^∗∗∗^	-0.35^∗∗∗^	-0.35^∗∗∗^
**Computational visual complexity**									
Edge density	0.41^∗∗∗^	0.39^∗∗∗^	0.46^∗∗∗^	0.52^∗∗∗^	3.88^∗∗∗^ (↑)	0.39^∗∗∗^	0.34^∗∗∗^	0.32^∗∗∗^	0.41^∗∗∗^
Feature congestion	0.47^∗∗∗^	46^∗∗∗^	0.53^∗∗∗^	0.59^∗∗∗^	3.86^∗∗∗^ (↑)	0.44^∗∗∗^	40^∗∗∗^	0.33^∗∗∗^	46^∗∗∗^
Subband entropy	0.20^∗∗∗^	0.16^∗∗∗^	0.21^∗∗∗^	25^∗∗∗^	2.07* (↑)	0.14^∗∗^	0.10^∗^	0.11^∗^	0.19^∗∗∗^

*For Experiment 4, *Z* is the statistical test of the difference in dependent correlations (Steiger, 1980). ^†^*p* < 0.10; ^∗^*p* < 0.05; ^∗∗^*p* < 0.01; ^∗∗∗^*p* < 0.001*.

Taken together, both of these results suggest that the ratings from Experiment 1 are valid and were not confounded by properties of the task design and provide evidence that the complexity ratings generalize across presentation times. In particular, Experiment 2 showed that the arousal-complexity bias is a robust phenomenon that was not caused by preferential processing of emotional arousing stimuli during limited presentation times or by a transfer from the preceding arousal and valence ratings.

## Experiment 3

In Experiment 1 we found that affective features, in particular emotional arousal, related to ratings of visual complexity beyond what could be explained by computational measures of visual complexity. While Experiment 2 replicated this result and ruled out important potential confounds, it did not shed any light on the mechanism by which this relationship with affect occurs. To further investigate the arousal-complexity bias, we chose to collect additional potentially mediating variables, i.e., cognitive and eye-tracking measures. Specifically, based on the higher semantic relatedness of emotionally arousing stimuli (Talmi and McGarry, 2012), we hypothesized that affective factors may increase the number of semantic concepts evoked when viewing a picture. For instance, a picture of a drug addict might evoke strong associations with concepts of disease, poverty, or crime, among other associations. These concepts may in turn inflate the experienced complexity which, might be falsely attributed then to visual complexity (also see Marin and Leder, 2013). We additionally hypothesized that affective factors may increase the number of eye fixations made when viewing the picture and the total fixation duration on the picture (Bradley et al., 2011; Ni et al., 2011). Furthermore, affective picture quality might increase the scan-path length (i.e., cumulative distance between fixations), which all in turn may inflate visual complexity ratings. To explicitly test for the mediator role of these additional variables we further conducted a path analysis as a means of directly describing the depending among the examined variables. This approach was done to holistically examine how each of the considered variables related to ratings of visual complexity for the pictures presented, and allowed us to isolate the more prominent relationships. For instance, if (a) feature congestion relates to the number of fixations and (b) both feature congestion and the number of fixations relate to visual complexity ratings, it is unclear how these each uniquely relate to ratings of visual complexity. Path analysis is appropriate for testing questions such as this (Norman and Streiner, 2003; Hooper et al., 2008; Shipley, 2016).

As trial length was further increased, by providing 30 s *after* each picture for generating semantic associates, we selected a random subset of 144 pictures from the previous 360 pictures used in Experiment 2. We again ensured that correlations between affective ratings, computational visual complexity, and visual complexity ratings were comparable to the whole stimulus set used in Experiment 1 (see **Table 1**).

### Methods

#### Participants

A total of 19 female volunteers with normal or corrected-to-normal vision participated. Consent and reimbursement procedures were identical to Experiment 1.

#### Materials

A subset of only 144 pictures from those used in Experiment 2 was selected. Again, pictures were selected randomly, but were based on the valence and topic categories to maximize coverage of the affective and visual complexity dimensions.

#### Apparatus

Stimuli were displayed on a 20-inch LCD monitor (Samsung SyncMaster 204B; display dimension = 40.64 cm × 30.48 cm; resolution = 1600 × 1200 pixels; refresh rate = 60 Hz). The eye-to-screen distance amounted to approximately 60 cm. Picture size was 900 × 600 pixels, thus amounting to a visual angle of 21.6° × 14.5°. An EyeLink 1000 eye-tracking system (SR Research Ltd., Ottawa, ON, Canada) was used to record eye movements. Monocular eye position data were sampled at 1000 Hz. Subjects’ heads were immobilized by a chin-rest. After blink detection, eye movement data were parsed into saccades and fixations using EyeLink’s standard parser configuration, which classifies an eye movement as a saccade when it exceeds 30°/s velocity or 8000°/s^2^ acceleration. Time intervals between saccades were defined as fixation.

### Procedure

Participants were told that they would need to list the ideas/concepts that came to mind after viewing each picture, hereafter referred to as “semantic associates.” On each trial, participants were presented with a fixation-cross overlaid on the mean luminance picture (i.e., the average of the 144 pictures across the RGB color dimensions) for 1000 ms, followed by the presentation of a picture for 5000 ms. Eye movements were also measured during this period of 6000 ms. Next, participants were presented with a blank screen with the number 0 on it, and told to press the “spacebar” key on the keyboard once for each semantic associate and to say the word out loud. Vocal responses were recorded using a computer microphone. The number on the computer screen incremented when the participant pressed the spacebar, to allow for visual feedback that the response had been recorded. Participants were given 30 s to list all of the associates. A 1000 ms inter-trial interval separated the trials.

The experiment consisted of 8 blocks of 18 pictures each, preceded by a buffer block of 9 pictures. The eye-tracker was calibrated prior to each block using a 9-point calibration procedure. The entire experimental session took approximately two and a half hours to complete.

#### Data Analysis

The number of semantic associates provided by each participant for each picture was scored as the number of “spacebar” presses. This key press measure was used, instead of the audio recordings, as the recordings were not audible for all participants (i.e., mumbling). Nonetheless, for participants who had usable audio recordings (*N = 13*), we conducted within-subject correlations and averaged them using Fisher’s *Z* transformation (Fisher, 1921; Corey et al., 1998) which we then refer to as *r_pop_*. The simplest expression of this transformation is *Z* = arctanh(*r*); see Corey et al. (1998) for a detailed discussion.

The key-press and scored-audio-recording measures were highly correlated [*r_pop_*(142, *N* = 13) = 0.92, *p* < 0.001, *Fisher’s Z* = 3.23] and yielded the same number of responses in 83% of the trials. When these measures disagreed, the key-press measure was more often the larger value [*M_MoreKey_* = 14%; *M_MoreV ocal_* = 3%; *t*(12) = 8.60, *p* < 0.001].

From the eye-tracking recordings, we quantified the number of fixations and the total fixation duration on each picture. Only fixations within the picture were counted and fixation coordinates were drift corrected relative to the initial fixation cross that preceded the picture onset. Furthermore, we calculated the scan-path length as the sum of all distances between individual fixations on each picture.

##### Path analysis

The path analysis was conducted to examine the relative relationships between affective ratings, computational measures, eyetracking measures, and semantic associates measure with ratings of visual complexity. Path analysis can be viewed as a specific type of structural equation modeling and is sometimes referred to as an analysis of covariance structures. In path analysis, several variables are designated as input variables for the path analysis, termed ‘exogenous’ variables. These are then used to explain covariances in other (endogenous) variables based on a theoretically defined structure of relationships between the variables.

**FIGURE 4:**
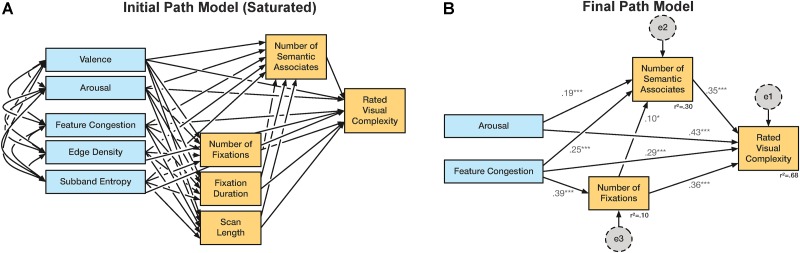
Path model used in Experiment 3. **(A)** Initial saturated path model. **(B)** Final path model. Variables shown in blue were coded as observed, exogenous variables; orange were observed, endogenous variables; gray were unobserved, exogenous variables (not shown in **A**). †*p* < 0.1; ^∗^*p* < 0.05; ^∗∗^*p* < 0.01; ^∗∗∗^*p* < 0.001.

In the analysis we began with several underlying assumptions: (1) Our dependent measure is ratings of visual complexity; models with any other variable as the final outcome measure were not considered. (2) The affective ratings and computational measures were our input (exogenous) measures. (3) The eyetracking and semantic associates measures were intermediate (endogenous) measures. (4) We considered all direct relationships between each measure and ratings of visual complexity. (5) We considered all indirect paths between each of the input and intermediate measures. (6) Eyetracking measures could also input the semantic associates measure. This initial structure is shown in **Figure 4A** and represents a fully ‘saturated’ model.

We first constructed the saturated path model using both affective ratings (arousal and valence) and all three computational measures (edge density, feature congestion, subband entropy) as observed, exogenous variables (**Figure 4A**). The additional measures obtained in Experiment 3 (number of semantic associates, number of fixations, fixation duration, and scanpath length) were observed; endogenous variables included as intermediate variables between the exogenous variables and the visual complexity rating. In this first, saturated model, all covariances between exogenous variables were included, and all paths, both direct and indirect, were included between the exogenous variables and visual complexity ratings. Unobserved, exogenous error terms were also included for all observed, endogenous variables. For the three rating measures (arousal, valence, and visual complexity), mean ratings were used from Experiment 1, as these were thought to be more representative as the pictures had been presented within the context of a large number of other pictures. Nonetheless, ratings from Experiments 2A and 2B were highly consistent with these ratings (see **Table 3**). The path analyses were conducted using IBM SPSS AMOS (Armonk, NY, United States).

After calculating this first model we removed covariances that were not found to be significant based on obtained critical ratios (covariance estimate/standard error; C.R. > 1.96). This procedure was then iteratively conducted to remove non-significant regression weights. Model fits at each iteration were also calculated through three measures: *χ^2^* (“badness-of-fit”; difference between observed data and model predictions), RMSEA (root mean square error of approximation), and AGFI (adjusted goodness-of-fit index) (see Hooper et al., 2008). Variables that were no longer connected to the other variables through covariances or regression weights were removed from the model.

### Results and Discussion

#### Correlation and Multiple Regression Analyses

All correlations are reported in **Table 4**. The number of semantic associates reported was highly correlated with ratings of visual complexity and arousal (based on ratings obtained in Experiment 1) and with two of the computational visual complexity measures (edge density and feature congestion). To test if the number of semantic associates reported was correlated to ratings of visual complexity beyond any effects that could be explained by the three computational measures of visual complexity, we conducted a partial correlation. Indeed, more semantic associates were reported for pictures that were rated higher in visual complexity [*r_p_*(139) = 0.48, *p* < 0.001]. This relationship of the higher number of evoked semantic associations and visual complexity ratings for arousing pictures supports the hypothesis that participant’s complexity ratings are inflated by associations triggered by arousing pictures.

**Table 4:** Correlations of measures obtained in Experiment 3 with ratings from Experiment 1 and computational visual complexity measures ^†^*p* < 0.10; ^∗^*p* < 0.05; ^∗∗^*p* < 0.01; ^∗∗∗^*p* < 0.001.

	*Ratings*			*Computational visual complexity*		
Measure	Visual complexity	Arousal	Valence	Edge density	Feature congestion	Subband entropy
No. of semantic associates	0.59 ^∗∗∗^	0.36 ^∗∗∗^	0.08	0.28 ^∗∗∗^	0.41 ^∗∗∗^	0.16^†^
**Eye movements**						
No. of fixations	0.51 ^∗∗∗^	0.03	-0.02	29 ^∗∗∗^	0.32 ^∗∗∗^	0.12
Fixation duration	0.03	-0.21 ^∗^	0.20 ^∗^	0.07	0.18 ^∗^	0.09
Scan-path length	0.24 ^∗∗^	-0.19 ^∗^	0.18 ^∗^	0.15 _†_	0.21 ^∗^	0.03

From the eye movement measures, both the number of fixations and scan-path length were correlated with visual complexity ratings. None of the eye movement measures correlated with subband entropy, but this is unsurprisingly considering the nature of subband entropy (i.e., it does not correspond to a spatial map, unlike edge density and feature congestion). Again we tested if these correlations with visual complexity ratings explained variability beyond that explained by the computational visual complexity measures. This partial correlation was significant for the number of fixations [*r_p_*(139) = 0.41, *p* < 0.001], but not the scan-path length [*r_p_*(139) = 0.12, *p* > 0.1]. However, as arousal was not correlated with the number of fixations and only negatively with scan path length these data suggest that arousal itself did not trigger a pattern of eye movements that results in higher complexity ratings.

Taken together, it appears that these additional measures, i.e., evoked semantic concepts and number of fixations, index processes that may serve as intermediates between basic visual processing (i.e., computational measures of visual complexity) and the ratings of visual complexity. To quantify how these additional variables improve our ability to explain variance in visual complexity ratings, we computed two additional multiple regression analyses: Our first model is identical to the last model from Experiment 1, but only included the subset of 144 pictures used in Experiment 3. As expected, this model performed similarly to the previous model, and was able to account for over half of the variance in visual complexity ratings [adjusted *R*^2^ = 0.58]. In our second model, we included only our four new measures (number of semantic associates, number of fixations, fixation duration, scan-path length) and were able to account for a large degree of variance [adjusted *R*^2^ = 0.46]. In our last model we included both sets of measures and obtained an adjusted *R*^2^ of 0.71.

Given the high number of correlations, we then sought to determine which variables contributed unique variance, and tested the causality of these relationships, using a path analysis. This analysis was conducted across all 144 pictures and a mediation structure was hypothesized between all of the variables of interest.

#### Path Analysis

We initial (saturated) path model is shown in **Figure 4A**, with relationships and variables iteratively removed if they were found to not significantly relate to the visual complexity ratings. After several iterations, we converged on the path analysis model shown in **Figure 4B**. All regression weights are significant, and the model as a whole was found to be a good fit to the data by all measures used [*χ^2^*(2,*N* = 144) = 0.21, *p* > 0.5; *RMSEA* < 0.001; *AGFI* = 0.996]. This final path model explained 68% of the variance in visual complexity ratings, nearly as much as the most complete multiple regression model [adjusted *R*^2^ = 0.71].

The only observed, exogenous variables retained were arousal and feature congestion, both of which had direct effects on visual complexity ratings, with standardized regression weights of 0.43 and 0.29, respectively. Both exogenous variables also had indirect effects on visual complexity ratings as well, 0.07 and 0.24, respectively. Of the indirect effects, feature congestion was found to significantly relate to both the number of semantic associates produced and the number of eye fixations, such that a picture that was more congested led to more associates and fixations. However, it is also important to consider that the measured eye movements were in response to a semantic associates instruction and have limited generalizability in relation to ratings of visual complexity or other instructions (e.g., see Yarbus, 1967). In particular, the task in this experiment differed substantially from the other experiments conducted here, which were based on ratings of visual complexity (and affective factors). Instead, the task of listing semantic associates is much more related to semantic memory and language processing, rather than visual processing. As such, participants likely engaged in this engaged in this task considerably differently than in the other experiments, e.g., participants likely scanned the pictures for cues to prompt semantic associations.

Taken together, as predicted arousing stimuli evoke more semantic associates and inflate visual complexity ratings partly via those. However, we found that arousing stimuli did not elicit more fixations, but rather were associated with shorter fixation durations and scan-path length. Additionally, the correlation between valence and eye movement measures suggests that subjects avoided looking on the highly arousing negative pictures. The number of fixations was correlated not only with visual complexity ratings but also with computational measures. In particular, feature congestion, but not edge density, led via more fixations to greater subject visual complexity. This suggests that finer details of the pictures led to a greater number of fixations and culminated in increased ratings of visual complexity; whereas edge density is a relatively more coarse index of picture’s computational visual complexity (e.g., see Rosenholtz et al., 2007). Importantly, the path model indicates a relatively strong, direct relationship between arousal and visual complexity ratings that was not mediated by semantic associates or fixations.

## Experiment 4

We have found robust evidence that affect has a direct relationship with visual complexity ratings. Specifically, Experiment 3 demonstrated that emotional arousal biases visual complexity ratings, without being mediated by factors such as semantic associates and number of fixations. This led us to two follow-up questions: (1) If made aware of this arousal-complexity bias, can participants consciously attenuate it? In other words, is the effect of arousal on perceived visual complexity accessible to introspection and cognitive control? (2) Does the magnitude of this arousal-complexity bias correlate with inter-individual differences in affect-related personality traits?

To test if people can consciously attenuate this arousal-complexity bias, in this experiment we had participants make two sets of visual complexity ratings. In the first set, participants were given the same instructions as in Experiments 1 and 2. In the second set, participants were informed that people’s ratings of visual complexity are also related to emotional properties of the pictures. The participants were then asked to try and focus on only the visual properties of the pictures when making their ratings. The primary analysis of interest was to examine how this instructional manipulation would influence the magnitude of the arousal-complexity relationship. Specifically, this was a test of the difference between two dependent correlations with one variable in common, which can be conducted as *Z*-test, following from Steiger (1980). As a validity check, ratings were also compared relative to those obtained in Experiment 1.

For this experiment, we recruited participants who had previously participated in a battery of questionnaires (see below). Of these questionnaires, we were interested in the trait anxiety measure from the Spielberger State-Trait Anxiety Inventory (STAI-T; Spielberger et al., 1983) and the Positive and Negative Affect Schedule (PANAS; Watson et al., 1988) as they are both commonly used as indexes of inter-individual differences in affective processing.

### Methods

#### Participants

A total of 40 female volunteers with normal or corrected-to-normal vision participated. Consent and reimbursement procedures were identical to Experiment 1.

In contrast to the prior and subsequent experiments, these volunteers had previously participated in a study that served the purpose to provide well-phenotyped participants for subsequent studies (for details see Kuhn et al., 2015). Briefly, participants were screened for psychiatric disorders (MINI diagnostic interview; Sheehan et al., 1998) prior to inclusion in the study, provided blood samples for genotyping, completed a battery of anxiety-related questionnaires, and agreed to be contacted for further imaging (fMRI) and behavioral studies. Our sample of 40 volunteers was randomly selected from this larger sample, though still had similar distribution characteristics (e.g., mean STAI-T in our sample was 36.2, mean in the Haaker et al., 2015, normative sample was 37.1). Prior to participation in this experiment, 30 of the participants had participated before at least in one fMRI study related to fear conditioning but had no experience to the pictorial stimuli used in the current experiment (Scharfenort and Lonsdorf, 2015; Kuhn et al., 2016).

#### Materials

The same subset of 360 pictures was used as in Experiment 2.

#### Procedure

The experiment consisted of three phases: (1) valence and arousal ratings, (2) visual complexity ratings, and (3) bias-aware visual complexity ratings. Before each phase, participants were given instructions explaining the task. The entire experimental session took approximately 2.5 h to complete.

##### Phase 1

Participants were presented with pictures, each followed by valence and arousal rating scales. Participants were given the same instructions as described in Experiment 1. On each trial, participants were presented with a fixation-cross overlaid on a mean luminance picture for 1000 ms, followed by the presentation of a picture for 5000 ms. Next, participants were sequentially shown the rating scales for valence and arousal (in a fixed order), with a 500 ms delay between the presentation of each rating screen. A 1000 ms inter-trial interval separated the trials. This was repeated for 360 trials. A block of 9 practice trials preceded the 360 trials, to familiarize participants to the task procedure, and were not included in the analyses.

##### Phase 2

Participants were presented pictures, each followed by a visual complexity rating scale (naïve instructions). The same sequence of presentations and timings were used as in the first phase apart from the instructions and scale. This was repeated for 180 trials, which were preceded by 9 practice trials.

##### Phase 3

Participants were told: “Sometimes people’s ratings of visual complexity are also related to the emotional properties of the picture. For this next set of ratings, try and focus on only the visual properties of the pictures, such as the number of edges, objects, symmetry, etc.” (bias-aware instructions). The importance of following this instruction was explicitly emphasized. To improve compliance with the instruction, and remind participants of this instructional manipulation, participants were additionally told: “Every so often, we will ask you how well you were able to follow the instructions. Here you will be asked to choose an option from the following scale: [Displayed a 9-point Likert scale with ‘1’ corresponding to ‘easy’ and ‘9’ corresponding to ‘hard’].” This compliance question, that assessed introspective and metacognitive abilities of the participants, was presented after every 30th trial. Apart from these additional instructions and compliance question, the procedure was identical to Phase 2.

Of the 360 pictures shown in Phase 1, 180 pictures were presented in Phase 2, and the remaining 180 pictures were presented in Phase 3. Across pairs of participants, pictures were pseudorandomly assigned to Phase 2 vs. Phase 3 such that pictures one participant saw in Phase 2, the other saw in Phase 3 (and vice versa).

### Results and Discussion

#### Can People Deliberately Attenuate the Arousal-Complexity Bias?

To test if our bias-aware instructions were able to attenuate the relationship between affect and visual complexity ratings, we correlated the visual complexity rating for each picture obtained with each instruction, with (a) the visual complexity ratings from Experiment 1, (b) the arousal and valence ratings from Phase 1 (of the current experiment), and (c) the computational measures of visual complexity previously calculated. While all of these correlations were significant (see **Table 3**), we also observe that the bias-aware correlations are attenuated for the affective ratings, but are increased for the computational measures of visual complexity. To directly compare these sets of correlations, we conducted *Z* tests that accounted for the dependent properties of these two correlations (see Steiger, 1980). As an additional confirmatory analysis, we calculated correlation and mean absolute difference for the arousal and valence ratings of from Phase 1 of the current experiment with the corresponding ratings of Experiment 1 [arousal: *r*(358) = 0.88, *p* < 0.001, *M_diff_* = 0.57; valence *r*(358) = 0.96, *p* < 0.001, *M_diff_* = 0.34].

Using the self-report compliance ratings as a measure of the participants’ metacognitive abilities regarding the bias-aware instruction, we found a large amount of inter-individual variance, where the participant with the lowest mean response on the 9-point Likert scale was 1.17, and the highest was 6 [*M(SD)* = 3.38 (1.28)]. Of the six compliance questions, we did not observe a change in responses given in the earlier trials vs. the later trials [*t*(39) = 0.31, *p* = 0.76].

To obtain a measure of the within-subject effect of the instructional manipulation, we calculated the slope of the relationship between the visual complexity and arousal ratings, for both instructions. Specifically, while a within-subject correlation is affected by both the slope and the spread of the data, we were particularly interested if the instructional manipulation modulated the coupling (slope) between the affective and complexity ratings. Indeed, the instructional manipulation (naïve vs. bias-aware instruction) did result in an attenuation of this slope [*t*(39) = 2.37, *p* = 0.02].

These results indicate that participants were able to deliberately attenuate the relationship between affect and visual complexity ratings (reduction in arousal-complexity bias). However, the large inter-individual variability in the compliance ratings suggests that participants differed substantially in this ability—at least subjectively—which will be discussed below. Importantly, the attenuation of the arousal-complexity bias was only nominal in magnitude and the correlation of arousal and visual complexity ratings was still significant under the bias-aware instruction. This suggests that the effect of arousal on visual complexity ratings is not fully within the domain of cognitive control; however, a limitation of this experiment is there may be an order effect, where people’s responses shift over the course of the experiment.

In an attempt to evaluate the potential influence of time or experience on the relationship between arousal and visual complexity ratings, we re-calculated the arousal-complexity correlation from Experiment 1, which occurred over two consecutive days, as four sequential segments of ratings: Day 1, first half; Day 1, second half; Day 2, first half; and Day 2, second half. Each of these segments consisted of 180 trials per participant. The arousal-complexity correlation for these four segments were [*r*(718) = 0.51, 0.50, 0.43, 0.44], respectively; in aggregate we observed *r* = 0.54 in Experiment 1. As such, we think it is likely that this relationship is relatively consistent within an experimental session, though there are several differences in the procedure between the current experiment and Experiment 1.

Surprisingly, however, the correlation between arousal and visual complexity—in the original, naïve instruction—was substantially lower in this experiment than in Experiments 1 and 2A, which we followed up in Experiment 5.

#### Relationship between Affective Processing and Personality Traits on Arousal-Complexity Bias

To test for a relationship between the measures of inter-individual differences in affective processing and personality traits, we conducted correlations using the slopes from the first set of ratings (naïve instructions). We found a non-significant correlations for the STAI-T [*r*(38) = 0.29, *p* = 0.07] and with both the positive and negative scales of the PANAS [both *p*’s > 0.1].

Taken together, the results of this experiment suggest that people are able to attenuate their bias in visual complexity ratings if they are made aware of it but only to a certain degree. In other words, a substantial part of the effect of arousal on experienced visual complexity is not accessible to introspection and cognitive control.

## Experiment 5

In all samples where both arousal and visual complexity ratings were obtained, we have observed significant positive correlations between the arousal and visual complexity ratings measures. In Experiments 1 and 2A, these correlations were strong [*r*’s of 0.54 and 0.55, respectively] (see **Tables 1**, **3**); however, in Experiment 4, this correlation was noticeably weaker, though still statistically significant [*r* = 0.27] (see **Table 3**). Interestingly, this occurred despite arousal and visual complexity ratings being highly consistent across experiments [all *r*’s > 0.9] (see **Table 3**). In Experiments 5A and 5B we aimed to test two hypothetical explanations of this lower correlation.

## Experiment 5A

One potential account of this reduced correlation is that in Experiment 4, the arousal and visual complexity ratings are separated by a delay (Phases 1 and 2), while in Experiments 1 and 2A the ratings are made sequentially. However, if this was critical, the visual complexity ratings from other participants with slightly different procedures (i.e., previous experiments reported in this paper) should also correlate more weakly with the arousal ratings made in Experiment 4, which was not the case [visual complexity ratings from Experiments 1, 2A, and 2B with arousal ratings from Experiment 4: all *r*’s between 0.46 and 0.50]. A second potential account for this reduced correlation is that unlike the prior experiments reported here, all pictures were presented twice in Experiment 4, once in Phase 1 (valence and arousal ratings) and then again in either Phase 2 or 3 (visual complexity ratings: naïve and bias-aware instructions, respectively). Thus, it is possible that this relationship between affect and visual complexity ratings may be related by the initial impact of the picture, and that re-presenting the picture attenuates this effect (e.g., habituation). Alternatively, people might rate visual complexity differently after they have processed the pictures previously because the pictures continued to be processed after the rating was made. Finally, the local context, e.g., the preceding pictures, may have had a carry-over effect, influencing the visual complexity ratings, but would have differed between the two presentations. This may have been particularly true if a picture was relatively dissimilar to its local context, such as presenting a negative picture within a set of neutral pictures. To test this possibility, here we manipulated whether participants either made ratings for the affective measures and visual complexity together, or if the ratings were made in separate blocks.

If this re-presentation is important, we additionally wondered how this effect may relate to explicit memory of the pictures. In other words, does this attenuation only occur if participants are able to remember seeing the picture previously in the experiment? This idea of a relationship between visual complexity and memory was suggested by Snodgrass and Vanderwart (1980), in regards to a potential relationship between visual complexity and stimulus recognition/novelty.

### Methods

#### Participants

A total of 22 female volunteers with normal or corrected-to-normal vision participated. Consent and reimbursement procedures were identical to Experiment 1.

#### Materials

The same subset of 360 pictures was used as in Experiment 2 and 4.

#### Procedure

The task consisted of four phases, completed sequentially.

##### Phase 1

Participants made valence and arousal ratings for 180 pictures. On each trial, participants were presented with a fixation-cross overlaid on a mean luminance picture for 1000 ms, followed by the presentation of a picture for 5000 ms. Next, participants were sequentially shown the rating scales for valence and arousal (in a fixed order), with a 500 ms delay between the presentation of each ratings screen. A 1000 ms inter-trial interval separated the trials. Therefore, this phase was equivalent to Phase 1 of Experiment 4.

##### Phase 2

Participants made valence, arousal, and visual complexity ratings for 90 additional pictures. Apart from the changes to the rating scales used, all phases had the same trial timings as in Phase 1. This phase was identical to the procedure used in Experiment 1 (apart from differences in the presentation duration and use of sequential ratings screens) and 2B.

##### Phase 3

Participants made visual complexity ratings for 90 pictures that were previously presented in Phase 1. This phase was matched to Phase 2 (naïve instruction) of Experiment 4.

##### Phase 4

Participants made visual complexity ratings, followed by a 6-point old/new-confidence rating for 180 pictures, 90 of which were from Phase 1 (i.e., the ‘old’ items). Ratings of 1 corresponded to ‘sure old’; ratings of 6 corresponded to ‘sure new.’

### Results and Discussion

As expected, arousal and valence were significantly correlated [Phase 1: *r*(358) = -0.47, *p* < 0.001; Phase 2: *r*(358) = -0.48, *p* < 0.001]. For comparison, this arousal-valence correlation from Experiment 2B was *r* = -0.53 for the same 360 pictures.

The correlation between arousal and visual complexity ratings was consistent with the prior experiments [Phase 2: *r*(358) = 0.45, *p* < 0.001]. For comparison, this arousal-complexity correlation from Experiment 2B was *r* = 0.55 for the same 360 pictures. Critically, this arousal-complexity correlation was not attenuated despite the pictures being presented for a second time for this visual complexity rating [Phase 3: *r*(358) = 0.48, *p* < 0.001; Phase 4 (old items): *r*(358) = 0.44, *p* < 0.001]. For comparison, this arousal-complexity correlation from Exp. 4 (naïve instructions) was *r* = 0.27 from the same 360 pictures. Thus, we did not observe an attenuated correlation due to the second presentation. (See **Table 3** for additional correlation measures.)

The memory responses indicate that participants did remember the earlier presentation of the picture. Of the old trials, 82.3% were rated as ‘sure old’; for the new trials, 64.8% were rated as ‘sure new’ (92.1% were any level of ‘new’ [rating of 3–6]). Thus, it appears that participants’ memory for the pictures was relatively good, despite the memory test being incidental. Calculating the arousal-complexity correlation for only the pictures correctly identified as ‘sure old’ did not indicate any difference in the strength of this relationship [*r*(358) = 0.42, *p* < 0.001]. Therefore, it does not appear that the visual complexity rating was differentially related to high confidently recognized and only familiar pictures. Moreover, this provides clear evidence that participants were able to episodically remember the initial presentation of the pictures.

In sum, it appears that our first alternative account that the attenuated correlations in Experiment 4 were due to the re-presentation of the picture did not bear out. However, Experiment 5A showed again how robust the effect of arousal on visual complexity ratings is as it was not reduced by the delay and repeated presentations.

## Experiment 5B

While the results of Experiment 5A provided a further replication of the relationship between affective and visual complexity ratings, they do not explain the attenuated correlations observed in Experiment 4. To explore this further, we sought finally to replicate the findings of Experiment 4 with a new sample of participants, based on the hypothesis that the participants of the former sample were self-selected to participate in studies related to emotional and aversive stimuli, as they had all participated in the previously described screening procedure and 75% of them also participated in at least in one fMRI study on fear conditioning (Scharfenort and Lonsdorf, 2015; Kuhn et al., 2016).

### Methods

#### Participants

A total of 12 female volunteers with normal or corrected-to-normal vision participated. Consent and reimbursement procedures were identical to Experiment 1. Only 12 volunteers were recruited because the main research question, i.e., the ability to consciously attenuate the arousal-complexity bias as well as the correlation with personality traits, had been assessed in Experiment 4. Experiment 5B was only intended to replicate the main finding, a higher correlation for arousal and visual complexity ratings than observed in Experiment 4.

#### Materials

The same subset of 360 pictures was used as in Experiments 2, 4, and 5.

#### Procedure

The procedure was identical to Experiment 4.

### Results and Discussion

We observed the correlation between arousal and visual complexity ratings to be significant with both instructions [naïve: *r*(358) = 0.50, *p* < 0.001; bias-aware: *r*(358) = 0.40, *p* < 0.001]. Though the correlation was attenuated with the bias-aware instructions, this effect was not significant [*Z* = 0.42, *p* = 0.67]. (See **Table 3** for additional correlation measures.) Nonetheless, the arousal-complexity relationship here was in-line with those found in our earlier experiments, and the change due to the different instructions was comparable to that of Experiment 4. While it is unclear why the effect was weaker overall in Experiment 4, this may be related to the high degree of familiarity with experimental settings and in particular aversive stimuli (e.g., electric shocks) of the volunteers that participated in Experiment 4.

## General Discussion

Across five experiments we found consistent evidence that ratings of visual complexity are related to affective properties of the stimuli. In particular, arousal, more than the valence, is related to higher ratings of visual complexity. On the contrary, various computational measures of visual complexity consistently indicate that there is no relationship between affective characteristics and computational measures of visual complexity, indicating a crucial difference in the stimuli properties captured by these ratings and computational measures. Importantly, these findings were robust across all experiments and were obtained using a large set of pictures.

Motivated by this initial finding, we conducted a number of successive follow-up experiments: In Experiment 2, we have shown that neither providing sufficient processing time to account for the preferential processing of arousing stimuli nor eliminating potential transfer effects form the preceding arousal rating reduces the correlation. Furthermore, Experiment 3 provided insight into the cognitive mechanisms of this effect, where we did find the number of semantic associates evoked by pictures to mediate this effect, i.e., arousing pictures evoke more semantic associates that in turn inflate the complexity rating. Importantly, this cognitive mechanisms explains only a rather small part of the arousal-complexity bias. In addition, we showed in Experiment 3 that arousal did not affect complexity ratings via influencing eye-movement patterns, as those were only related to computational measures of visual complexity. Critically, the path analysis is compatible with a relatively strong, direct relationship between arousal and visual complexity ratings. In Experiment 4, we showed that the arousal-complexity bias could be attenuated if volunteers are made aware of this bias. Experiment 5A rejected a potential mediatory role of habituation or other effect of repeated presentations and explicit memory on the arousal-complexity bias. Finally, Experiment 5B suggested that volunteers that are very familiar with experimental paradigms involving highly aversive stimuli, as fear conditioning, may show a reduced arousal-complexity bias due to these prior experiences, though further research into inter-individual differences is necessary (e.g., see Bies et al., 2016; Güçlütürk et al., 2016; Street et al., 2016). In sum, the arousal-complexity bias is a robust phenomenon, which is consciously accessible only to a small, anxiety-related degree. Arousal relates to visual complexity ratings only weakly via the cognitive factor evoked semantic associations and not via inducing differences in visual exploration.

This leads to the question: how does arousal affect visual complexity ratings? Ratings of visual complexity are based on visual features present in the object but are influenced also by our knowledge, experience, and understanding of the visual object (Machado et al., 2015). In addition, rating the perceived visual complexity of a picture on a Likert scale is a meta-cognitive process where volunteers must reflect on the quality of their perception. As the ratings were always made only after the picture presentation, and in some experiments only after preceding arousal and valance ratings, volunteers either need to sample the necessary information for the rating and keep them in working memory until the rating was done, or keep the picture in visual working memory and base the rating on this mnemonic representation. Therefore, arousal may directly bias visual processes, bias perceived complexity via cognitive—for instance, prior knowledge based—processes that influence the perception, or affect meta-cognitive processes of the rating itself. Future research may need to manipulate the pictures themselves to explicitly test the causal directionality of this affect-complexity relationship. Systematic manipulation of picture stimuli needs to be done with careful consideration, as changing colors, blurring, or otherwise processing the pictures may make them less ecologically valid.

The assessment of the evoked semantic associates in Experiment 3 as well as the bias-aware instruction in Experiment 4 were attempts to address potential effects of arousal on the higher cognitive, constructive processes of perception and meta-cognition. The results of both experiments suggest a limited role of incidental triggered semantic associations and (meta-) cognitive process that are consciously accessible. This does not rule out that perceiving arousing pictures might differ with respect to other cognitive processes which influences are difficult to control. For instance, arousing pictures could be more effectively activate prior knowledge in terms of schemata or scripts that could be in addition more detailed and more complex (Ghosh et al., 2014). Such activated schemata may influence the perceived complexity and would not necessarily be fully captured by the assessment of semantic associates. However, in the Deese–Roediger–McDermott (DRM) illusion or false memory paradigm that is sometimes used to address schema activation arousal results in fewer errors in addition to better memory for details but not gist (Storbeck, 2013; Van Damme, 2013). Somehow related, arousal might also increase the amount of inference and hypotheses triggered by a picture in order to understand its meaning which is part of the perceptual process (Machado et al., 2015). These additional cognitive processes could be misattributed during the rating to reflect higher ratings of visual complexity. However, one would expect that both, stronger activation of more detailed and complex schemata and evoking more complex hypotheses, would be reflected also in the semantic associations which we assessed in Experiment 3. Taken together, the current experiments do not provide evidence that arousal relates to visual complexity ratings mainly via acting on (meta-)cognitive processes related to perception or the rating procedure.

On the other hand, it is well known that emotional arousal relates to basic visual processes (Pourtois et al., 2013; Markovic et al., 2014; Mather et al., 2016). In particular, it has been argued that arousal enhances the signal-to-noise ratio and vividness of perception, emotional arousing stimuli attract more selective attention and are preferential processed (Mather et al., 2016). In addition, emotional arousing pictures are perceived and remembered more vividly (Markovic et al., 2014). Consistent with these effects of arousal on visual processing, a number of fMRI and EEG studies have found greater brain activity in the visual cortex for affective pictures (e.g., Schupp et al., 2003; Bradley et al., 2007; Herrmann et al., 2008; Olofsson et al., 2008; Todd et al., 2012; Simola et al., 2013). These literature would be consistent with the interpretation that the relationship between arousal and the meta-cognitive visual complexity ratings is based on its effects on visual processing and perception.

Finally, the described arousal-complexity bias may also have practical implications. When studying the influence of affect on cognitive processes such as attention and memory, it is important to control for other stimulus properties that may confound the manipulation of interest. Visual complexity is one such picture property, however, studies that have matched picture sets for complexity have often relied on ratings to assess a picture’s complexity. The results of the experiments presented here clearly indicate that ratings of visual complexity are not only indexing computational characteristics of pictorial stimuli, but are also related to affective factors. The current data demonstrates that the distinction between ratings and computational measures of visual complexity is critical and must be carefully considered when it is appropriate to use either approach. Specifically, when the research question is, for instance, related to basic processing of arousal or valence, the researcher should control for computational visual complexity to remove this feature as a potential confound of brain activity and eye movements. The results of Experiment 1 show that the combination of the three formal measures of computational measures used here (edge density, feature congestion, subband entropy) provide distinct estimates of visual complexity (though other computational measures may also be useful, e.g., see Braun et al., 2013). However, if the researcher is interested in other stimulus properties (e.g., category membership such as faces vs. houses), it may instead be preferred to match the picture sets for differences in visual complexity ratings as these also incorporate affective factors to some degree. Recently it was suggested that in such a situation a combination of computational measures can replace the time consuming normative rating of visual complexity (Machado et al., 2015). Our data show that this can be only done when the stimulus material is neutral with respect to valence and arousal. In other cases, the researcher may want to deliberately manipulate visual complexity across conditions (e.g., Nguyen and McDaniel, 2015), and care should be taken to ensure that the resulting picture sets are different based on only the dimension desired to be manipulated, visual complexity.

## Ethics Statement

This study was carried out in accordance with the recommendations of Board of Physicians, Hamburg, Germany with written informed consent from all subjects. All subjects gave written informed consent in accordance with the Declaration of Helsinki. The protocol was approved by the Board of Physicians, Hamburg, Germany.

## Author Contributions

CM and TS conceived the overall study design. All authors contributed to the experimental design and manuscript writing. CM and JB programmed the experiments. CM analyzed the data.

## Conflict of Interest Statement

The authors declare that the research was conducted in the absence of any commercial or financial relationships that could be construed as a potential conflict of interest. The reviewer AC and handling Editor declared their shared affiliation.

## Footnotes

1 Here we converted the pictures to CIELab 1976 color space (designed to mimic the responses of the human eye) and then computed the Canny edge detection on the L^∗^ dimension using the lower and upper thresholds suggested by Rosenholtz et al. (2007), 0.11 and 0.27, respectively.
